# The Effect of Flavored E-cigarettes on Murine Allergic Airways Disease

**DOI:** 10.1038/s41598-019-50223-y

**Published:** 2019-09-20

**Authors:** David G. Chapman, Dylan T. Casey, Jennifer L. Ather, Minara Aliyeva, Nirav Daphtary, Karolyn G. Lahue, Jos L. van der Velden, Yvonne M. W. Janssen-Heininger, Charles G. Irvin

**Affiliations:** 1 Department of Medicine, University of Vermont College of Medicine, Burlington, Vermont United States; 2 Department of Pathology, University of Vermont College of Medicine, Burlington, Vermont United States; 3 Translational Airways Group, School of Life Sciences, University of Technology Sydney, Ultimo, NSW Australia; 4 Emphysema Centre, Woolcock Institute of Medical Research, Sydney, NSW Australia

**Keywords:** Asthma, Experimental models of disease, Translational research

## Abstract

Flavored e-cigarettes are preferred by the majority of users yet their potential toxicity is unknown. Therefore our aim was to determine the effect of selected flavored e-cigarettes, with or without nicotine, on allergic airways disease in mice. Balb/c mice were challenged with PBS or house dust mite (HDM) (Days 0, 7, 14–18) and exposed to room air or e-cigarette aerosol for 30 min twice daily, 6 days/week from Days 0–18 (n = 8–12/group). Mice were exposed to Room Air, vehicle control (50%VG/%50PG), Black Licorice, Kola, Banana Pudding or Cinnacide without or with 12 mg/mL nicotine. Mice were assessed at 72 hours after the final HDM challenge. Compared to mice challenged with HDM and exposed to Room Air, nicotine-free Cinnacide reduced airway inflammation (p = 0.045) and increased peripheral airway hyperresponsiveness (p = 0.02), nicotine-free Banana Pudding increased soluble lung collagen (p = 0.049), with a trend towards increased airway inflammation with nicotine-free Black Licorice exposure (p = 0.089). In contrast, all e-cigarettes containing nicotine suppressed airway inflammation (p < 0.001 for all) but did not alter airway hyperresponsiveness or airway remodeling. Flavored e-cigarettes without nicotine had significant but heterogeneous effects on features of allergic airways disease. This suggests that some flavored e-cigarettes may alter asthma pathophysiology even when used without nicotine.

## Introduction

Electronic cigarette (e-cigarette) use has dramatically increased in recent years due to its portrayal as a healthier alternative to tobacco cigarettes. E-cigarette use amongst high school aged children in the US jumped from 1.5% in 2011 to over 15% in 2015^1^. Similarly, almost 9% of 18–24 year olds are current e-cigarette users^2^. E-cigarettes administer nicotine as an aerosol, do not require tobacco combustion and therefore are assumed to remove the risks associated with tobacco cigarettes. However, e-cigarettes contain numerous toxic compounds long associated with adverse respiratory outcomes^3^. The humectant propylene glycol leads to respiratory symptoms when inhaled^4^, while nicotine adversely effects respiratory health^5^. E-cigarette liquids are also available in a seemingly limitless combination of flavor additives, with almost all users under 30 and almost 70% of older adults using flavored e-cigarettes^6^. Additionally, up to 66% of US high school aged children use e-cigarettes with only flavoring/s i.e. no nicotine^7^. However, e-cigarette flavorings have been shown to have toxic effects on human airway epithelial and immune cells^8–11^. Despite the high use of flavored e-cigarettes and the potential toxicity, little is known as to the adverse effects of flavored e-cigarettes on lung function and respiratory health.

Asthmatics are particularly susceptible to the effects of toxic, inhaled substances such as tobacco, with tobacco smoking asthmatics reporting worse asthma control, more unscheduled health care visits, and a greater requirement for oral corticosteroids^12^. This increased disease severity is due to both worse lung function^13^ and greater severity of airway hyperresponsiveness^14^. There is currently little evidence as to the effect of e-cigarette use in patients with asthma. One study reported that asthmatic patients who transitioned from tobacco cigarette smoking to e-cigarette use reported improved asthma symptoms and lung function^15^. However, this does not address whether e-cigarettes themselves may be detrimental to asthmatics. In healthy volunteers, acute e-cigarette use can reduce lung function^16–18^. Importantly, the reduction in lung function was exaggerated in patients with asthma^18^, suggesting that asthmatics may be more susceptible to the effects of e-cigarettes. However, it is unknown whether these acute effects of e-cigarettes on lung function reflect the potential effects on asthma pathophysiology.

**Figure 1:**
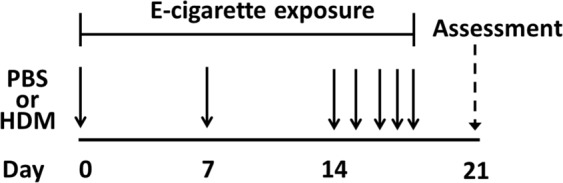
Schematic representation of the induction of allergic airways disease with concomitant exposure to e-cigarette vapor. Mice were administered intranasal house dust mite (HDM) or phosphate buffered saline (PBS) on days 0, 7 and 14–18. Starting on day 0 and continuing through day 18, mice were exposed to e-cigarette aerosol for 30 min twice/day, 6 days/week. Mice were evaluated on day 21, 72 hours following the final HDM/PBS instillation.

Since e-cigarettes contain numerous toxic compounds and reduce lung function following acute use, our objective was to determine the effect of flavored e-cigarettes on the development and severity of allergic airways disease (Fig. 1). To determine whether any effects were due to nicotine, we compared the effect of different flavored e-cigarettes with nicotine and without nicotine. A nicotine concentration of 12 mg/mL was chosen based upon reported use patterns amongst daily e-cigarette users when the study was developed^19,20^. E-cigarette flavors were chosen based on the *in vitro* toxicity of cinnamon flavors^21^ (Kola and Cinnacide) and creamy/buttery flavors^8,9^ (Banana Pudding). Black Licorice was chosen as a flavor distinct from the aforementioned flavors.

## Results

### Effect of e-cigarette exposure on weight

The effect of e-cigarette exposure on weight gain was assessed by measuring weight on Day 21 before the assessment of lung function (Supplementary Fig. 1). As sex is a determinant of body weight, data from female and male mice were analyzed separately. There was no effect of HDM on weight in female or male mice exposed to e-cigarette with or without nicotine (p ≥ 0.2 for all). When combining both PBS and HDM groups, there was no difference in weight between female mice exposed to Room Air and any of the e-cigarettes. In contrast, male mice exposed to PG/VG, Black Licorice and Cinnacide with nicotine had reduced weight compared to Room Air exposed mice (p < 0.05 for all). Similarly, there was a trend towards reduced weight in mice exposed nicotine-free Kola compared to Room Air (p = 0.065).

### Effect of e-cigarette exposure on HDM-induced airway inflammation

#### Flavored e-cigarettes without nicotine

**Figure 2:**
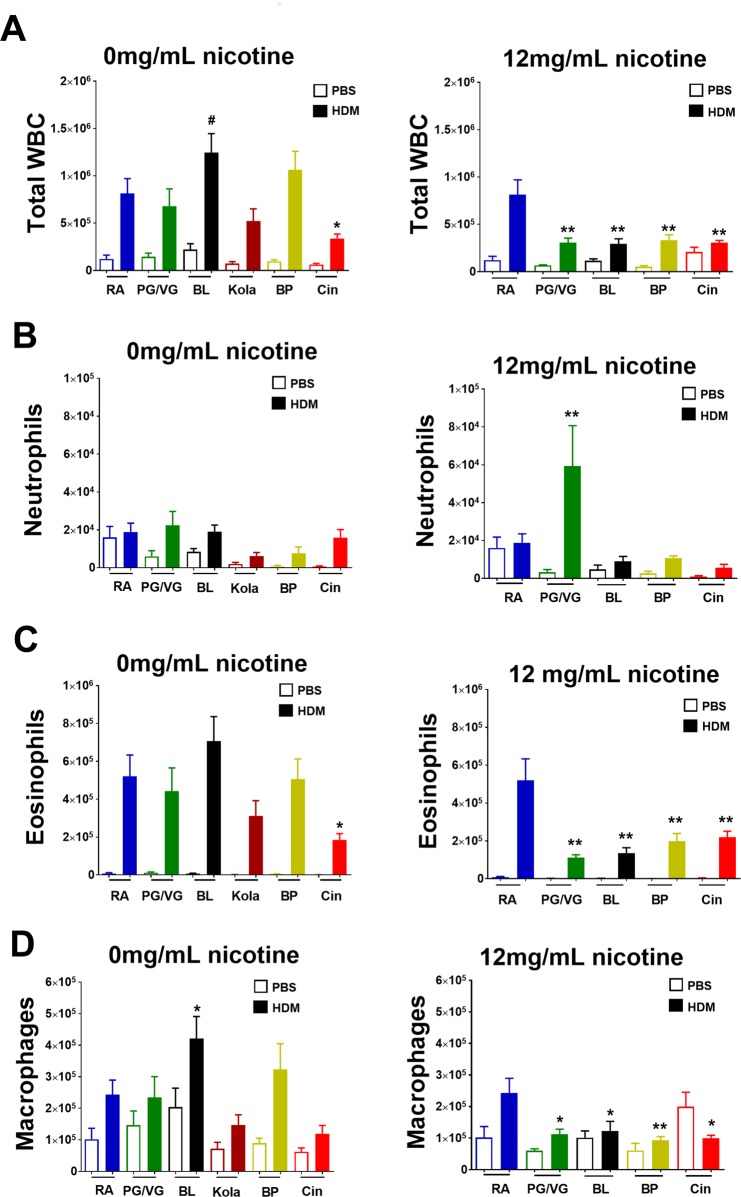
The effect of electronic cigarettes without and with nicotine on airway inflammation in allergic airways disease. Airway inflammation measured in bronchoalveolar lavage fluid from mice challenged with phosphate buffered saline (control) or house dust mite (HDM) and exposed to Room Air (RA, blue), 50% propylene glycol/50 %vegetable glycerin (PG/VG, vehicle, green), Black Licorice (BL, black), Kola (dark red), Banana Pudding (BP, yellow) or Cinnacide (Cin, red). Mice were exposed to e-cigarette liquid without nicotine (left hand panels, 0 mg/mL) or e-cigarette liquid containing 12 mg/mL nicotine (right hand panels). Shown are (**A**) total leukocyte cell counts, (**B**) eosinophils, (**C**) neutrophils and (**D**) macrophages. Data are presented as mean ± SEM of 6–12 mice/group. In mice exposed to nicotine-free e-cigarette, HDM increased total leukocytes, eosinophils, neutrophils and macrophages (HDM effect p < 0.001 for all). In mice exposed to e-cigarette with nicotine, HDM increased total leukocytes (p < 0.001), eosinophils (p < 0.001) and neutrophils (p < 0.002) but did not alter macrophages (HDM effect p = 0.16). Comparisons between HDM treated mice exposed to Room Air HDM and exposed to e-cigarettes were made by Dunnett analyses: *p < 0.05, **p < 0.01 and ^#^p < 0.09.

To determine the effect of nicotine-free e-cigarette exposure on allergic airways inflammation following HDM we measured inflammatory cells in BALF (Fig. 2, left hand panels). Total leukocytes, eosinophils, neutrophils and macrophages in BALF were all increased with HDM (p < 0.0001 for all). However, total leukocytes and eosinophils were reduced in HDM mice exposed to Cinnacide without nicotine compared to Room Air (p = 0.045 and 0.02, respectively). In contrast, macrophages were increased in HDM mice exposed Black Licorice compared to Room Air (p = 0.05), with a trend towards increased total leukocytes (p = 0.089). PG/VG, Kola or Banana Pudding did not alter total leukocytes, eosinophils or macrophrages. Similarly, there was no difference in neutrophils between HDM mice exposed to Room Air and those exposed to any of the nicotine-free e-cigarettes. This suggests that flavored e-cigarettes without nicotine have variable effects on airway inflammation during allergic airways disease, with the direction of the effect and the type of inflammatory cells affected dependent upon the specific flavor.

#### Flavored e-cigarettes with nicotine

As shown in Fig. 2 (right hand panels), all e-cigarettes containing 12 mg/mL nicotine dampened airway inflammation. Compared to HDM mice exposed to Room Air, mice exposed to PG/VG and all flavored e-cigarettes had reduced total leukocytes (p < 0.001 for all), eosinophils (p < 0.001 for all) and macrophages (p < 0.05 for all). In contrast, while there was no difference between HDM mice exposed to Room Air and any of the flavored e-cigarettes (p > 0.5 for all), there was an increase in neutrophils in HDM mice exposed to PG/VG compared to Room Air (p < 0.001).

Importantly, the allergic airways disease in the present model was predominantly eosinophilic and the percentage of eosinophils was largely unaltered by e-cigarette exposure (Supplementary Fig. 2). However, there was a small skewing towards a reduced proportion of eosinophils in HDM mice exposed to VG/PG with nicotine and Black Licorice with nicotine compared to Room Air HDM (p < 0.01 and 0.045, respectively). Representative H&E stained images of the extent of inflammatory cells surrounding the airway is shown in Supplementary Fig. 3. Taken together, the present findings suggest that the effect of flavored e-cigarettes without nicotine on HDM-induced airway inflammation is dependent upon the specific flavor. In contrast, nicotine universally suppressed airway inflammation regardless of the flavor.

### Effect of e-cigarettes on baseline lung function

Lung function was measured at baseline before the methacholine challenge. In mice exposed to e-cigarettes without nicotine, HDM treatment increased baseline Rrs, Rn, G and H (p < 0.05 for all) but did not alter Ers (p = 0.25). However, there was no difference in any measure of airway mechanics between HDM treated mice exposed to Room Air and any of the e-cigarette exposures (p > 0.5 for all post-hoc comparisons). Similarly, in mice exposed to e-cigarettes with 12 mg/mL nicotine, HDM treatment increased AHR as measured by Rrs, Rn, G and H (p < 0.05 for all) but not by Ers (p = 0.51). Again, none of the nicotine e-cigarette exposures had an effect on any measure of baseline airway mechanics (p > 0.4 for all).

### Effect of e-cigarettes on HDM-induced airway hyperresponsiveness

#### Flavored e-cigarettes without nicotine

**Figure 3:**
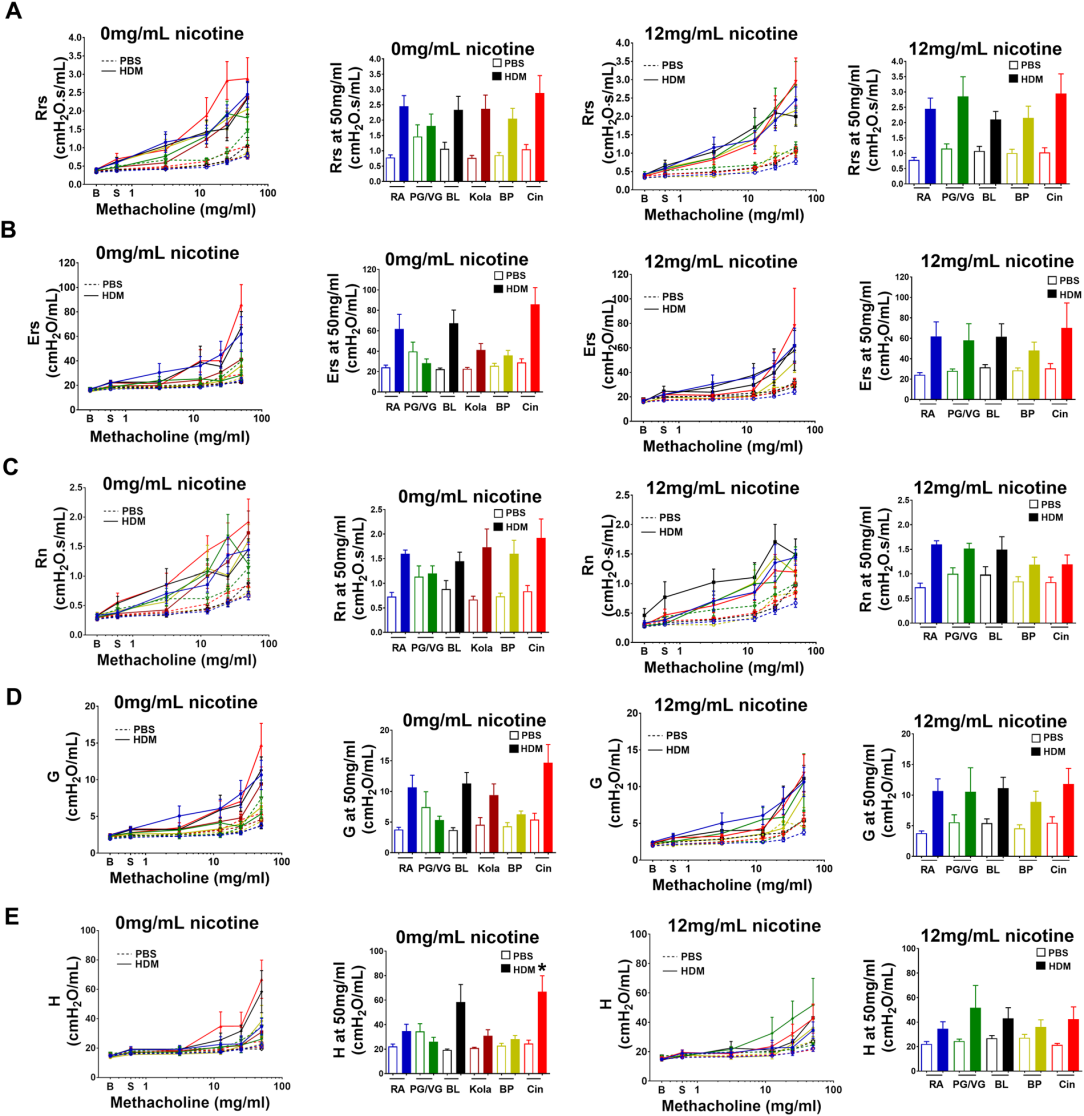
The effect of electronic cigarettes without and with nicotine on the severity of airway hyperresponsiveness in allergic airways disease. Airway hyperresponsiveness to methacholine was measured by: (**A**) respiratory system resistance (Rrs); (**B**) respiratory system elastance (Ers); (**C**) Newtonian resistance (Rn); (**D**) tissue damping (G); (**E**) tissue elastance (H). Mice were challenged with phosphate buffered saline (PBS control, dotted line) or HDM (solid line) and exposed to Room Air (RA, blue), 50% propylene glycol/50% vegetable glycerin (PG/VG, vehicle, green), Black Licorice (BL, black), Kola (dark red), Banana Pudding (BP, yellow) or Cinnacide (Cin, red). Mice were exposed to e-cigarette liquid without nicotine (left hand panels, 0 mg/mL) or e-cigarette liquid containing 12 mg/mL nicotine (right hand panels). Parameters of airway mechanics were measured at baseline (B), after saline control (S) and after each dose of methacholine (3.1, 12.5, 25 and 50 mg/mL). The response to methacholine at each dose was quantified as the average of the three peak measurements for each parameter. Full dose-response curves and the response at 50 mg/mL methacholine are shown. Data are presented as mean ± SEM of 6–12 mice/group. In mice exposed to nicotine-free e-cigarette, HDM increased Rrs, Ers, Rn, G and H (HDM effect p < 0.001 for all). In mice exposed to e-cigarette with nicotine, HDM increased Rrs, Ers, Rn, G and H (HDM effect p < 0.001 for all). Comparisons between HDM treated mice exposed to Room Air HDM and exposed to e-cigarettes were made by Dunnett analyses: *p < 0.05.

The effect of e-cigarettes on AHR was determined by measurement of changes in lung function during a methacholine challenge (Fig. 3). In mice exposed to e-cigarettes without nicotine, HDM treatment increased AHR as measured by Rrs, Ers, Rn, G and H (p < 0.001 for all). There was no difference between HDM treated mice exposed to Room Air and any of the e-cigarette exposures as measured by Rrs (p > 0.8 for all), Ers (p > 0.1 for all), Rn (p > 0.5 for all) and G (p > 0.15 for all) at the highest dose of methacholine. However, AHR measured by H at the highest dose of methacholine was increased in HDM mice exposed to Cinnacide without nicotine compared to Room Air HDM (p = 0.02). There was no difference in H at 50 mg/mL methacholine between the other e-cigarette exposures and Room Air (p > 0.1 for all).

#### Flavored e-cigarettes with nicotine

In mice exposed to 12 mg/mL nicotine, HDM treatment increased AHR as measured by Rrs, Ers, Rn, G and H (p < 0.001 for all). However, there was no difference between HDM treated mice exposed to Room Air and any of the e-cigarette exposures as measured by Rrs (p > 0.8 for all), Ers (p > 0.9 for all), Rn (p > 0.8 for all), G (p > 0.9 for all) and H (p > 0.5 for all) at the highest dose of methacholine.

### Effect of e-cigarette exposure on HDM-induced airway remodeling

**Figure 4:**
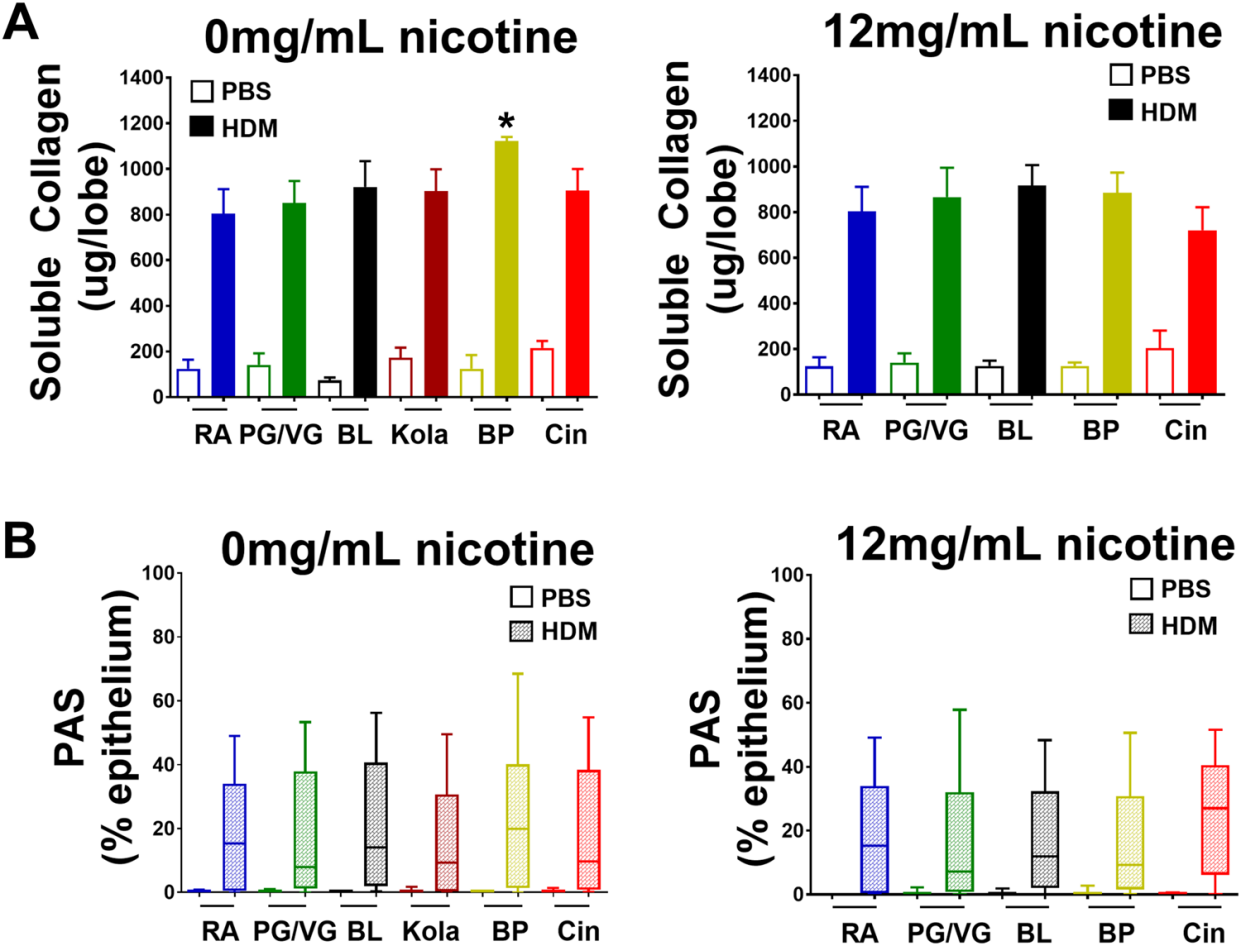
The effect of electronic cigarettes without and with nicotine airway remodeling in allergic airways disease. Mice were challenged with phosphate buffered saline (control) or HDM and exposed to Room Air (RA, blue), 50% propylene glycol/50% vegetable glycerin (PG/VG, vehicle, green), Black Licorice (BL, black), Kola (dark red), Banana Pudding (BP, yellow) or Cinnacide (Cin, red). Mice were exposed to e-cigarette liquid without nicotine (left hand panels, 0 mg/mL) or e-cigarette liquid containing 12 mg/mL nicotine (right hand panels). Airway remodeling was measured as (**A**) soluble collagen (**B**) the percentage of Periodic Acid-Schiff (PAS) staining within the airway epithelial layer. Data are presented as mean ± SEM or Box-and-whisker plots of 6–12 mice/group. In mice exposed to nicotine-free e-cigarette, HDM increased soluble collagen and PAS staining (HDM effect p < 0.001 for all). In mice exposed to e-cigarette with nicotine, HDM increased soluble collagen and PAS staining (HDM effect p < 0.001 for all). Comparisons between HDM treated mice exposed to Room Air HDM and exposed to e-cigarettes were made by Dunnett analyses: *p < 0.05.

We determined the effect of e-cigarettes on airway remodeling through measurement of soluble lung collagen content and histological assessment of mucous hyperplasia from PAS stained lung tissue (Fig. 4 and Supplementary Fig. 4). In mice exposed to nicotine-free e-cigarettes, HDM treatment increased soluble content (p < 0.0001), with the amount of soluble collagen increased in mice exposed to Banana Pudding compared to Room Air exposure (0.049). There was no difference between HDM treated mice exposed to Room Air and the other nicotine-free e-cigarettes (p > 0.9 for all). In contrast, despite an increase in airway epithelial PAS staining with HDM treatment (p < 0.0001), there was no difference between HDM treated mice exposed to Room Air and any of the e-cigarettes without nicotine (p > 0.9 for all).

In mice exposed to 12 mg/mL nicotine, soluble lung collagen was increased with HDM treatment (p < 0.0001) but there was no difference between mice exposed to Room Air and any of the e-cigarettes (p > 0.9 for all). Similarly, HDM treatment increased airway epithelial PAS staining (p < 0.0001) but there was no difference in PAS staining between HDM treated mice exposed to Room Air (p > 0.45 for all). Representative PAS stained images are shown in Supplementary Fig. 3.

## Discussion

This is the first study to determine the effects of flavored e-cigarettes with and without nicotine on allergic airways disease. Our findings suggest that flavored e-cigarettes without nicotine can alter allergic airways disease but that the effect is dependent upon the specific flavor. Indeed, the effect of nicotine-free e-cigarette exposure varied greatly between the flavors: Cinnacide suppressed airway inflammation and increased peripheral AHR, Banana Pudding increased soluble collagen content, and there was a trend towards exaggerated airway inflammation in mice exposed to Black Licorice. On the other hand, nicotine universally dampened airway inflammation independent of flavor with no effect on AHR or airway remodeling. These findings suggest that flavored e-cigarettes have the potential to alter the pathophysiology associated with allergic airways disease, but that the direction and extent of the effect is dependent upon exposure to a specific flavor.

Our findings highlight the variable effect of flavored e-cigarettes on airway inflammation and that the suppression caused by nicotine is independent of flavor. In the present study Black Licorice exaggerated airway inflammation whereas Cinnacide caused suppression. Several studies have reported varied *in vitro* toxicity of flavored e-cigarettes on a variety of respiratory and non-respiratory cell types^8–11^. Consistent with our findings regarding Cinnacide, Clapp *et al*.^9^ reported suppressed immune responses in human alveolar macrophages and neutrophils exposed to cinnamon flavored e-cigarettes. The authors subsequently replicated these findings using the flavor additive cinnamaldehyde leading them to conclude that cinnamaldehyde was detrimental to immune cell function. This is consistent with the anti-inflammatory effect of cinnamaldehyde in a mouse model of cardiac inflammation^22^. Although it is likely that Cinnacide used in the present study contained cinnamaldehye, this was not confirmed and therefore we can only speculate on its role in the current findings.

In contrast to nicotine-free e-cigarette exposure, there was a ubiquitous suppression of airway inflammation with e-cigarette exposure containing nicotine. Nicotine is known to have anti-inflammatory properties^23,24^ and findings with tobacco smoke exposure suggest that reduced airway inflammation is due to abnormal eosinophil migration into the airways^25^. Reduced eosinophilia in mice with allergic airways disease exposure to e-cigarette containing nicotine is consistent with reduced eosinophilia in asthmatics who smoke tobacco cigarettes compared to asthmatic non-smokers^26^. Furthermore, a recent study reported that e-cigarette vapor condensate led to alveolar macrophage apoptosis and necrosis, both of which occurred to a greater extent when the exposure contained nicotine^27^. Alveolar macrophages are crucial to innate immunity^28^ and suppression of macrophage immune function by e-cigarettes would be expected to compromise the immune response to infection. In addition, wide-spread suppression of immune-related gene expression has been reported in nasal epithelial cells from e-cigarette users^29^. The airway epithelium is also crucial in coordinating the innate immune response to infection^30^ with abnormal airway epithelial responses correlated with the severity of viral exacerbations in asthma^31^. Indeed, there is growing *in vitro* and mouse model evidence that e-cigarette use is associated with impaired anti-bacterial and anti-viral responses^32–34^. Although exposure to Cinnacide without nicotine suppressed airway inflammation in the present study, it is unclear whether this reflects the potential to contribute to an impaired response to infection. However, recent findings of reduced airway ciliary function *in vitro*^35^ strengthen the speculation that cinnamon-flavored e-cigarettes may contribute to immune dysfunction.

There is currently a lack of evidence in both animal models and human data on the effect of e-cigarettes on lung function. In the present study, AHR was increased in mice exposed to Cinnacide without nicotine, despite having reduced airway inflammation. Cinnamaldehye, the characteristic component of cinnamon flavoring, is known to activate transient receptor potential ankyrin 1 (TRPA1) in the upper airways leading to cough^36^. However, this would be expected to increase AHR measured by central airway narrowing (Rn) and not tissue elastance, as reported in the present study. The reduction in *in vitro* ciliary function in response to cinnamon flavored e-cigarette exposure^37^ would be expected to reduce mucus clearance. Mucus production during methacholine challenge is thought to contribute to AHR in allergic airways disease via increased airway closure, as measured by tissue elastance (H)^38^. Therefore, an impairment in mucus clearance would lead to increased airway closure during methacholine challenge and increased AHR as measured by H, as seen in the present study following exposure to the Cinnacide e-cigarette flavor.

There was no effect of exposure to the vehicle humectant PG/VG or e-cigarettes containing nicotine on AHR. However, we utilized a murine model of allergic airways disease that involves relatively short-term exposure to e-cigarette aerosol. Two previous studies have measured the effect of long-term e-cigarette exposure on airway mechanics and AHR in non-allergic mice. Larcombe^39^ reported worse baseline mechanics in mice exposed to tobacco flavored e-cig with 100% VG or 100% PG for 8 weeks, with and without nicotine. Furthermore, exposure to 100% VG increased AHR, again regardless of the presence of nicotine, while 100% PG had no effect. In contrast, Garcia-Arcos^40^ reported increased AHR following 16 weeks of exposure to nebulized e-cigarette liquid containing nicotine (50% PG/VG) but not nicotine-free liquid. Importantly, the two studies differ in respect to heating of the liquid, since Garcia-Arcos *et al*.^40^ nebulized the liquid whereas Larcombe *et al*.^39^ used a commercial e-cigarette device. Therefore these findings may reveal that long-term nicotine inhalation promotes AHR whereas the development of AHR due to vegetable glycerin requires both long-term exposure and heating of the liquid. This is possibly related to the production of toxic aldehydes and/or acrolein caused by heating of vegetable glycerin^41^ or metal nanoparticles emitted from device itself when heated^42^.

There is a similar lack of evidence on the effect of e-cigarettes on lung function in humans. Thirty minutes of nicotine-free e-cigarette use (70% PG/30% VG) was shown to have no effect on spirometric or forced oscillation technique measurements in healthy or asthmatic non-smokers^43^. Similarly, Flourish *et al*.^44^ found no effect of 30 minutes of e-cigarettes containing nicotine and tobacco flavor (>60% PG) on spirometry in healthy tobacco smokers. In contrast, Ferrari *et al*.^16^ reported that five minutes of using nicotine-free e-cigarettes containing hazelnut flavor additives reduced PEF and FEV_1_ in tobacco smokers but not non-smokers. Vardavas *et al*.^17^ also reported that 5 min e-cigarette use using liquid containing nicotine and tobacco flavor (>60% PG) in healthy tobacco smokers increased respiratory system resistance but did not alter reactance. Recently, Lappas *et al*.^18^ reported that 10 puffs of an e-cigarette containing nicotine and tobacco flavor worsened respiratory system resistance and reactance in healthy subjects and mild asthmatics. These effects were exaggerated in the asthmatics but had resolved to baseline within 15 min in both groups. Although there are several differences between these human studies, it was the three studies of acute use (5 min or 10 puffs) that reported abnormal lung function whereas the longer studies did not (30 min). It is therefore attractive to speculate that initial inhalation of e-cigarette aerosol induces bronchoconstriction which subsides with continued use. Indeed, one study reported such a time-dependent effect of e-cigarette use on cough reflex sensitivity^45^. However, the contribution of sustained, long-term use on the development of abnormal lung function and AHR remains to be elucidated.

Exposure to Banana Pudding without nicotine increased soluble lung collagen content in mice with allergic airways disease. In contrast, there was no effect of the other nicotine-free flavored e-cigarettes and all e-cigarette exposures containing nicotine. Similarly, a previous study found no effect of e-cigarette aerosol containing nicotine on lung fibrosis in non-allergic mice^46^. This may suggest that airway remodeling is linked to specific flavor additives rather than nicotine. For example, diacetyl (2,3-butanedione) is found in numerous common flavors such^47^ as “buttery/creamy” and fruit flavors, and has been detected in many flavored e-cigarette liquids^48^. Diacetyl and related chemicals cause lung fibrosis in rodents^49^ and led to bronchiolitis obliterans in workers at a microwave-popcorn plant^50^, highlighting the potential for flavored e-cigarettes to contribute to lung fibrosis. Additionally, Crotty-Alexander *et al*.^51^ reported renal, hepatic and cardiac fibrosis in mice following long-term (3/6 months) exposure to non-flavored e-cigarette aerosol containing nicotine suggesting the relationship between e-cigarette exposure and fibrosis may differ across different organs.

Although the present study does not directly compare to tobacco exposure, findings from several tobacco exposure models allow indirect comparison. In the present study, the effect of e-cigarettes with nicotine was confined to suppression of allergic airway inflammation, with no effect on AHR or features of airway remodeling. Lancaster *et al*.^52^ induced allergic airways disease over 2.5 weeks with tobacco cigarette smoke exposure throughout. Cigarette smoke exaggerated allergic airways inflammation, due to effects on eosinophils and neutrophils, increased AHR and increased mucous hyperplasia. Using an identical study design, Kumar *et al*.^53^ reported that tobacco cigarette smoke increased HDM-induced airway eosinophils, neutrophils and mucous metaplasia, although effects on AHR did not reach significance. Similarly, concurrent exposure to ovalbumin and tobacco cigarette smoke contributes to more severe allergic airways inflammation, due to effects on eosinophils and neutrophils, and AHR^54^. These findings of increased airway inflammation, AHR and features of airway remodeling in tobacco models similar to the present study may suggest that e-cigarettes are less likely to worsen asthma pathophysiology than tobacco cigarette smoking. However, the present study used a mild exposure regime when compared to those used for tobacco exposure. In our study, mice were exposed to e-cigarettes for 30 min twice/day whereas Lanckacker *et al*.^52^ and Moerloose *et al*.^54^ used four exposures/day, and Kumar *et al*.^53^ used three exposures/day. Therefore, it is possible that the lack of effect of e-cigarettes with nicotine on AHR and airway remodeling in the present study is due to reduced exposure compared to these tobacco mouse models. Nonetheless, a potential reduction in harm with e-cigarettes compared to tobacco cigarettes is consistent with the reduction in the extent and number of toxic particles in e-cigarette aerosol compared to tobacco smoke^55^. This speculation of reduced harm with e-cigarettes is strengthened by the single report of improved lung function, AHR and symptoms in asthmatic tobacco smokers who transition to e-cigarettes^15^.

The present study evaluated several different flavored e-cigarettes in a well-described animal model of allergic disease; however, there are a few limitations that should be mentioned. Firstly, liquids were purchased from different companies who are likely to have different manufacturing practices. The effect of e-cigarettes containing nicotine on airway inflammation was the same for all liquids suggesting minimal effects of any differences in manufacturing procedures. However, there was a small difference in the percentage of PG and VG between the liquids, with control and Black Licorice used at 55%/45% while the other liquids were 50%/50%. The combination of flavor aldehydes and PG produces PG acetals, with the amount measured dependent upon PG content i.e. 70% PG > 50% PG > 30% PG^56^. However, it is unknown whether PG acetal formation is greater with 55% PG compared to 50% PG, and whether this could lead to the increased airway inflammation measured in mice exposed to Black Licorice. Lastly, while we endeavored to use the same commercial liquid with and without nicotine, we were unable to obtain the Atomic Cinnacide flavor without nicotine due to discontinuation by the manufacturer. We therefore utilized a similarly strong cinnamon flavor based on consumer reviews (Cinnacide). Given that both e-cigarettes liquids dampened airway inflammation, the difference in manufacturer did not appear to have a significant effect on the overall findings.

To assess the potential toxicity of flavored e-cigarette to patients with asthma the present study investigated the effects of various flavored e-cigarettes with and without nicotine on a mouse model of allergic airways disease. In this model of mild exposure, e-cigarettes with and without nicotine had substantially different effects. Depending upon the specific flavor, e-cigarettes without nicotine enhanced or suppressed airway inflammation, increased airway hyperresponsiveness and increased features of airway remodeling. In contrast, there was a consistent suppression of airway inflammation without any effect on AHR or airway remodelling due to e-cigarettes with nicotine, regardless of the flavor. These findings highlight the potential for flavored e-cigarettes, in the absence of nicotine, to negatively contribute to asthma outcomes and warrant caution in promoting their use to patients with asthma. Future investigation into the individual chemical constituents of e-cigarettes driving these toxic effects may provide important insight for the regulation of e-cigarettes.

## Materials and Methods

### Study design

All experiments were approved by the Institutional Animal Care and Use Committee at the University of Vermont (#15-022). Experiments were conducted in accordance with the Public Health Service policy on ‘Humane Care and Use of Laboratory Animals’ and the Assessment and Accreditation of Laboratory Animal Care guidelines. Balb/c mice were purchased from Jackson Laboratories (Bar Harbor, ME, USA) and studied at eight weeks of age. Equal numbers of male and female were included in each group, with 6–10mice/PBS control group and 8–12/HDM group. Mice were administered 50 ug of intranasal HDM (Greer Laboratories, Lenoir, NC, USA) in sterile PBS (1 mg protein/mL) or PBS alone on 7 occasions over three weeks: on days 0, 7 and 14–18 (Fig. 1). Mice were evaluated 72 hours following the final instillation.

Mice were exposed for 30 min twice/day, 6 days/week consistent with total daily e-cigarette exposure times of previous studies^34,39,40^. Exposures started at Day 0 before the first administration of HDM/PBS and continued until Day 18 following the last administration of HDM/PBS. On days 0, 7 and 14–18, at least 60 min was left between e-cigarette exposure and HDM/PBS administration, and between HDM/PBS administration and the second daily e-cigarette exposure. For exposures, mice were placed in a Perspex box (W × L × H = 25.4 × 17.8 × 20.3 cm) connected to a MasterFlex® peristaltic pump (Cole Palmer, Vernon Hills, IL, USA) set a 1 L/min. Room Air exposure involved continuously running the pump when disconnected from the e-cigarette device. For e-cigarette exposure, aerosols were produced using a variable temperature control eVic-VT e-cigarette (Joyetech USA, Tustin, CA, USA) using a titanium coil (0.4Ω) set at 550 °F with a limit of 45 W. The e-cigarette device was actuated for 4 s every 60 s to reflect human use^57^, with room air continuously pumped between actuations. Separate e-cigarette atomizers were used for each flavor and nicotine concentration. Separate exposure chambers were used for 0 mg/mL and 12 mg/mL nicotine exposures and chambers were thoroughly cleaned after each exposure.

Mice were exposed to room air or one of the following e-cigarette liquids; vehicle control (50% vegetable glycerin/%50 propylene glycol, PG/VG), Black Licorice, Kola, Banana Pudding and Cinnacide (Table 1). Liquids contained either 0 mg/ml or 12 mg/ml nicotine for each liquid.

**Table 1:** Details of the e-cigarette liquids used for exposure.

Commercial name	Humectant	Nicotine	Company
Control	50% PG/50% VG	0 ml/mL	Vapor Girl, Chapel Hill, NC, USA
Control	50% PG/50% VG	12 ml/mL	Vapor Girl, Chapel Hill, NC, USA
Black Licorice	50% PG/50% VG	0 mg/mL	Tasty Vapor, Oakland, CA, USA
Kola	55% PG/45% VG	0 mg/mL	Vapor Girl, Chapel Hill, NC, USA
Banana Pudding	55% PG/45% VG	0 mg/mL	Vapor Girl, Chapel Hill, NC, USA
Cinnacide	55% PG/45% VG	0 mg/mL	The Vapor Depot, Ponderay, ID, USA
Black Licorice	50% PG/50% VG	12 mg/mL	Tasty Vapor, Oakland, CA, USA
Banana Pudding	55% PG/45% VG	12 mg/mL	Vapor Girl, Chapel Hill, NC, USA
Atomic Cinnacide*	55% PG/45% VG	12 mg/mL	Dodgy Brothers, Malaysia

PG = propylene glycol, VG = vegetable glycerin *E-cigarette was produced by Tasty Vapor but purchased from a secondary source after Tasty Vapor discontinued production.

### Assessment of airway mechanics and airway hyperresponsiveness (AHR)

Mice were anesthetized with intraperitoneal pentobarbital sodium (90 mg/kg), tracheotomized, and mechanically ventilated at 200 breaths/min with a tidal volume of 0.25 mL and positive end-expiratory pressure of 3 cmH_2_O (FlexiVent, SCIREQ, QC, Canada). Airway mechanics were assessed by the forced oscillation technique in which respiratory impedance was partitioned into respiratory system resistance and respiratory system elastance, a measure of the stiffness of the lung. Airway mechanics were also measured by input impedance in which the Constant Phase Model^58^ provides measurements of Newtonian resistance (Rn), a measure of airway resistance, tissue damping (G), a measure of tissue resistance and tissue elastance (H), a measure of lung stiffness. Airway responsiveness was assessed during airway challenge with increasing doses of aerosolized methacholine (saline control, 3.125, 12.5, 25 and 50 mg/ml). The response to methacholine at each dose was quantified as the average of the three consecutive peak measurements.

### Collection and analysis of bronchoalveolar lavage

Lungs were lavaged with 1.0 ml of PBS and centrifuged at 1200 × g for 5 minutes to isolate cells. Total cell counts were performed using the Countess Automated Cell Counter (Invitrogen, Waltham, MA). Differential cell counts were performed using the Hema3 kit (Fisher Scientific, Kalamazoo, MI) by counting 300 cells per mouse.

### Measurement of collagen content

Newly deposited lung collagen was measured in the upper right lobe using the Sircol Assay (Biocolor, UK) after overnight digestion with 10 mg/ml pepsin in 0.5 M acetic acid as directed by the manufacturer.

### Histopathology

Lungs were inflated to 30 cmH_2_O and fixed with 4% paraformaldehyde in PBS followed by paraffin embedding. Paraffin blocks were cut into 5 µm sections and mounted to slides. Inflammatory cell infiltration in the lung was confirmed from hematoxylin and eosin stained sections. Mucus metaplasia was assessed from lung sections stained with periodic-acid Schiff reagent. Five bronchiolar airways (x40 magnification) were imaged from each slide and the percentage of PAS staining within the epithelial layer was calculated using Metamorph image analysis software (Molecular Devices, Sunnyvale, CA).

### Statistical analysis

The effects of e-cigarettes were evaluated by linear least squares regression using effect parameters for HDM challenge, e-cigarette exposure and an interaction between the two (JMP^®^ Pro 10, SAS Institute Inc., Cary, NC, USA). Subsequent comparisons between HDM treated mice exposed to Room Air and e-cigarettes were performed using Dunnett post-hoc analyses. PAS staining data were analyzed by repeat-measures to account for multiple airways from each individual lung slice. Separate analyses were performed for e-cigarettes exposures with (12 mg/mL) and without nicotine (0 mg/mL) but the same Room Air control data were used for both analyses. Data are presented as mean ± SEM or median ± IQR and p values < 0.05 were regarded as statistically significant.

## Supplementary information

Supplementary File
