# Supplementary material for: The Effect of Flavored E-cigarettes on Murine Allergic Airways Disease
Source: Sci Rep. 2019 Sep 20;9:13671. doi: 10.1038/s41598-019-50223-y (PMC6754426; doi:10.1038/s41598-019-50223-y)
Supplement: Supplementary file 1 — Supplementary File [file 41598_2019_50223_MOESM1_ESM.docx]

**Supplementary Material**

**The Effect of Flavored E-cigarettes on Murine Allergic Airways Disease**

David G. Chapman^1,3,4^,Ph.D, Dylan T. Casey^2^, BS, Jennifer L Ather ^1^, PhD, Minara Aliyeva^1^, MD, Nirav Daphtary^1^, MS, Karolyn G Lahue^2^, Jos L. Van der Velden^2^, Ph.D, Yvonne MW Janssen-Heininger^2^, Ph.D, Charles G. Irvin^1^, Ph.D

**
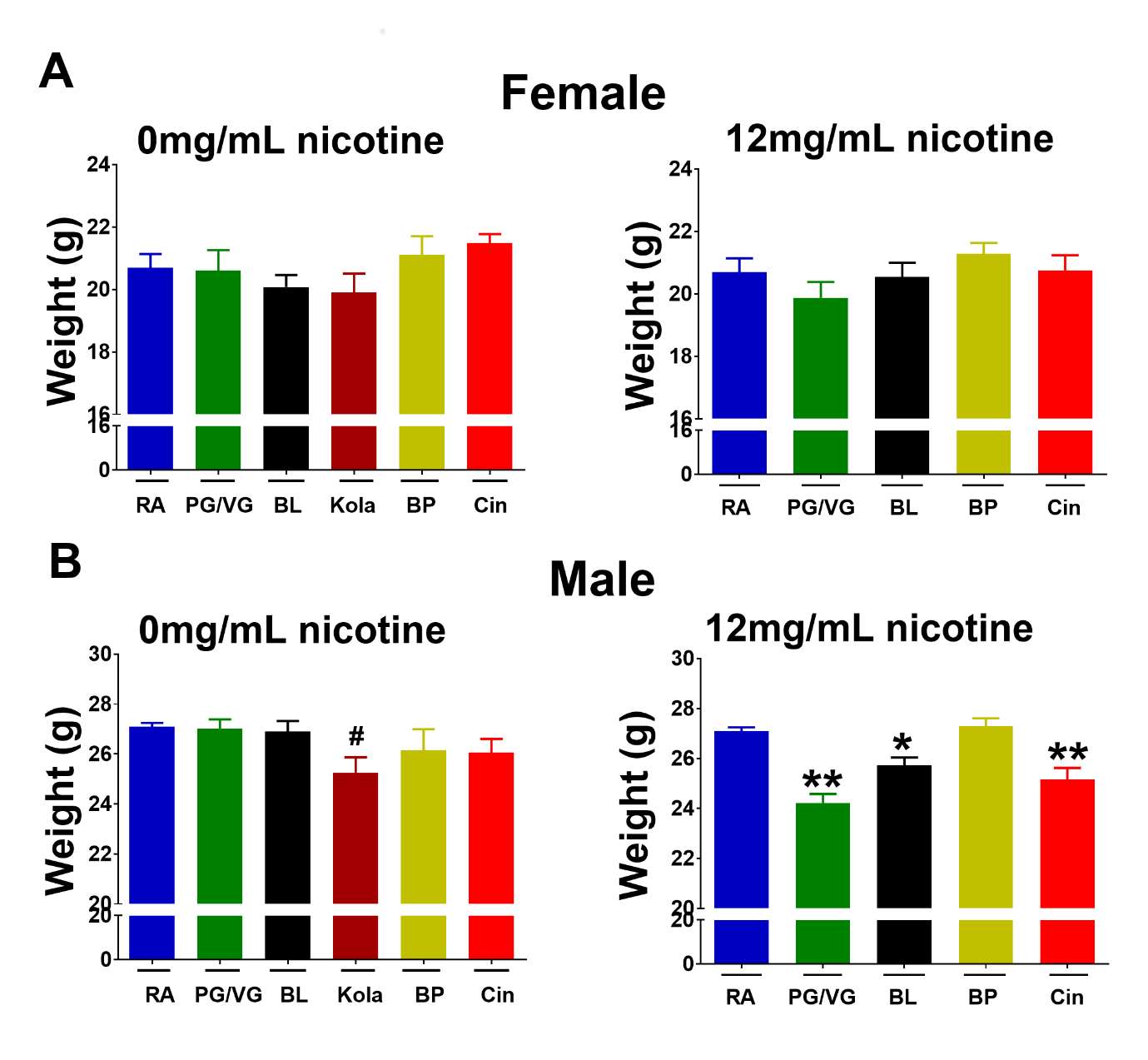
**

**Supplementary Figure 1: The effect of electronic cigarettes without and with nicotine on weight.** Mice were challenged with phosphate buffered saline (PBS, control) or house dust mite (HDM) and exposed to Room Air (RA), 50%propylene glycol/50%vegetable glycerin (PG/VG, vehicle), Black Licorice (BL), Kola, Banana Pudding (BP) or Cinnacide (Cin). Mice were exposed to e-cigarette liquid without nicotine (left hand panels, 0mg/mL) or e-cigarette liquid containing 12mg/mL nicotine (right hand panels). Data are presented as mean ± SEM of 6-10 mice/group. Female (A) and male mice (B) were analysed separately but as there was no effect of HDM in either sex with or without nicotine (p ≥ 0.2 for all), PBS and HDM mice were combined for these analyses. Comparisons between mice exposed to Room Air HDM and exposed to e-cigarettes were made by Dunnett analyses: **p < 0.001, *p < 0.05, ^#^p = 0.065.

**
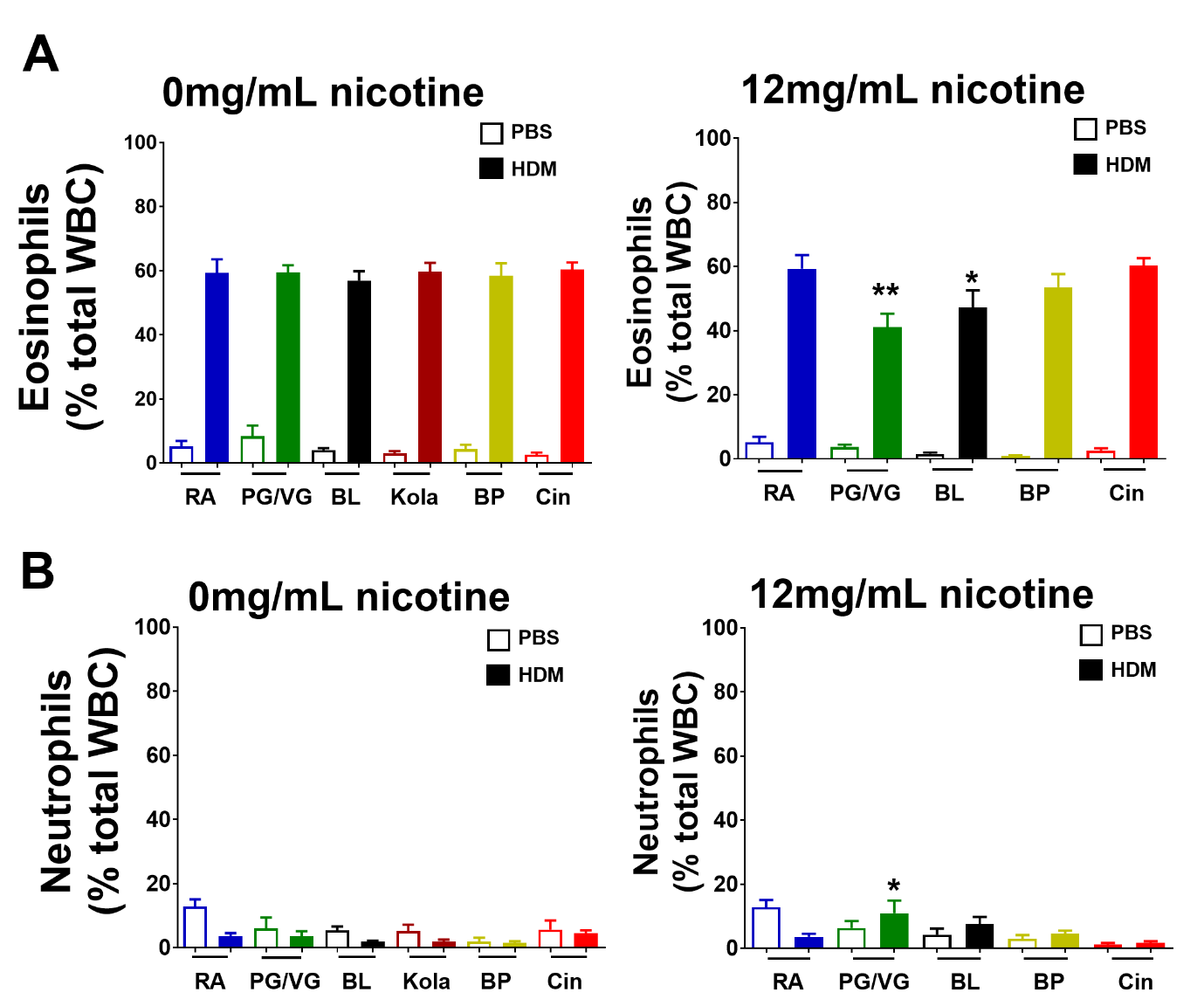
**

**Supplementary Figure 2: The effect of electronic cigarettes without or with nicotine on the airway inflammatory phenotype of HDM-induced allergic airways inflammation.** Airway inflammation measured in bronchoalveolar lavage fluid from mice challenged with phosphate buffered saline (control) or house dust mite (HDM) and exposed to Room Air (RA, blue), 50%propylene glycol/50%vegetable glycerin (PG/VG, vehicle, green), Black Licorice (BL, black), Kola (dark red), Banana Pudding (BP, yellow) or Cinnacide (Cin, red). Mice were exposed to e-cigarette liquid without nicotine (left hand panels, 0mg/mL) or e-cigarette liquid containing 12mg/mL nicotine (right hand panels). Shown are (A) eosinophils as a percent of total cells and (B) neutrophils as a percent of total cells. Data are presented as mean ± SEM of 6-12 mice/group. In mice exposed to nicotine-free e-cigarette, HDM increased % eosinophils and reduced % neutrophils (HDM effect p<0.001 for both). In mice exposed to e-cigarette with nicotine, HDM increased % eosinophils (p<0.001 for all) but did not affect % neutrophils (p=0.65). Comparisons between HDM treated mice exposed to Room Air HDM and exposed to e-cigarettes were made by Dunnett analyses: *p < 0.05, **p < 0.01.

**
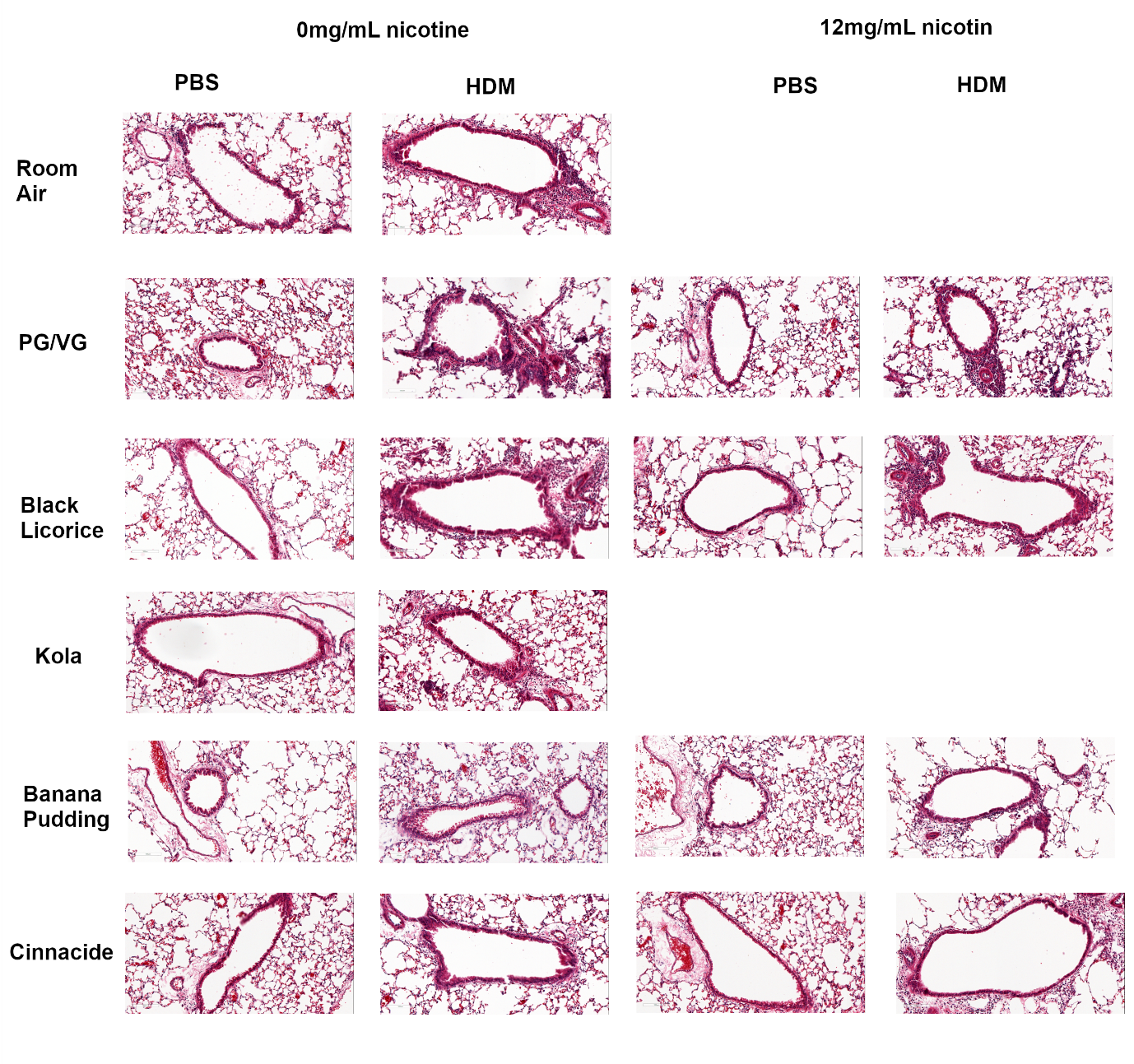
**

**Supplementary Figure 3: Hematoxylin and eosin stained lung slices from mice exposed to electronic cigarettes without and with nicotine.** Mice were challenged with phosphate buffered saline (PBS, control) or house dust mite (HDM) and exposed to Room Air (RA), 50%propylene glycol/50%vegetable glycerin (PG/VG, vehicle), Black Licorice (BL), Kola, Banana Pudding (BP) or Cinnacide (Cin). Mice were exposed to e-cigarette liquid without nicotine (left hand panels, 0mg/mL) or e-cigarette liquid containing 12mg/mL nicotine (right hand panels). Representative images were selected based on the median total BAL leukocyte count from each group. Images were taken at 20x magnification.

**
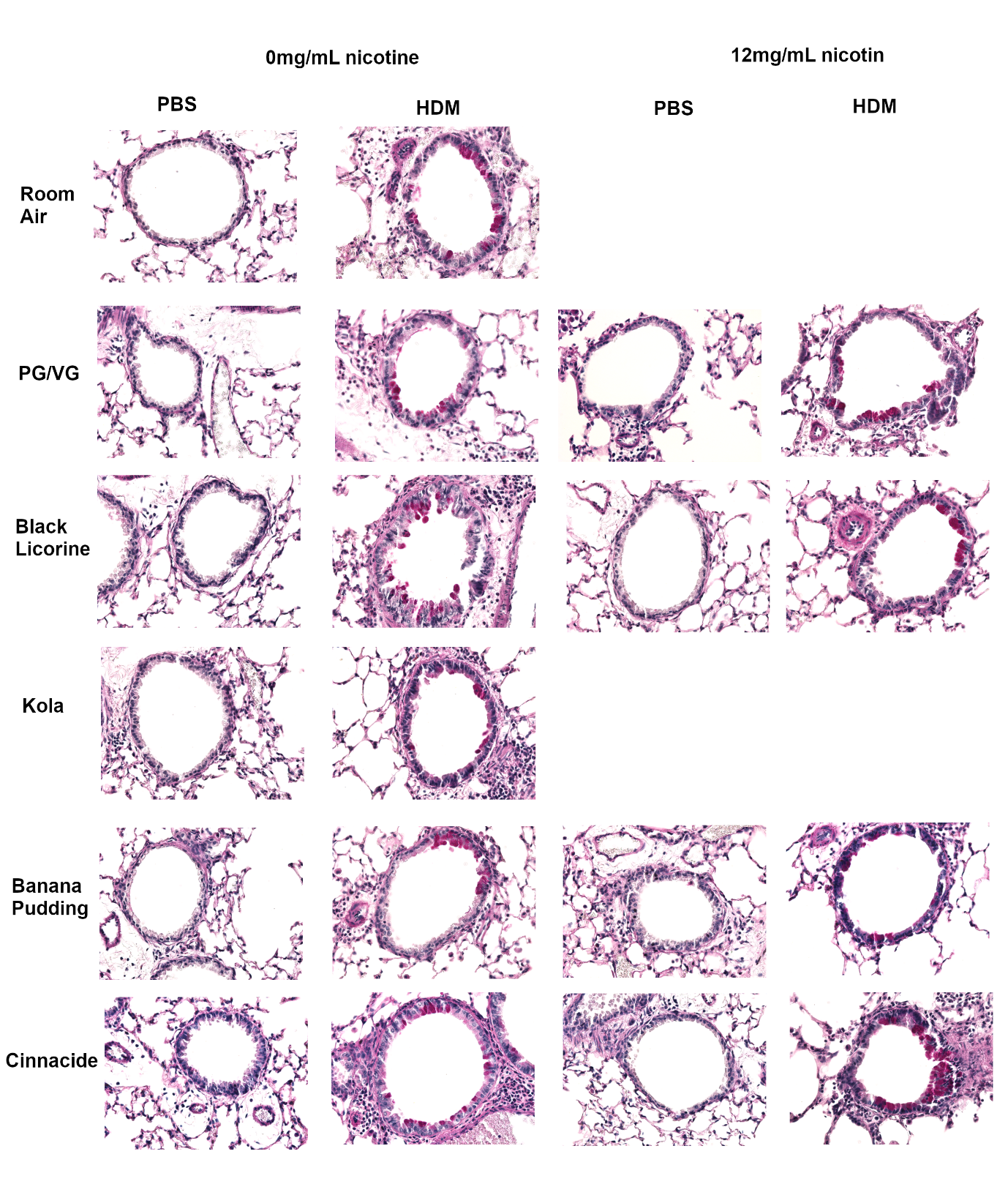
Supplementary Figure 4: Periodic acid-Schiff stained lungs from mice exposed to electronic cigarettes without and with nicotine.** Mice were challenged with phosphate buffered saline (PBS, control) or house dust mite (HDM) and exposed to Room Air (RA), 50%propylene glycol/50%vegetable glycerin (PG/VG, vehicle), Black Licorice (BL), Kola, Banana Pudding (BP) or Cinnacide (Cin). Mice were exposed to e-cigarette liquid without nicotine (left hand panels, 0mg/mL) or e-cigarette liquid containing 12mg/mL nicotine (right hand panels). Representative images were selected based on the median percentage of PAS staining within the epithelial layer from each group.
